# *In situ* nanoscale mapping of electrical and catalytic properties

**DOI:** 10.1093/nsr/nwae202

**Published:** 2024-06-12

**Authors:** Christopher S Kley

**Affiliations:** Helmholtz Young Investigator Group Nanoscale Operando CO_2_ Photo-Electrocatalysis, Helmholtz-Zentrum Berlin für Materialien und Energie GmbH, Germany; Department of Interface Science, Fritz Haber Institute of the Max Planck Society, Germany

By enabling the conversion of sustainably-derived electrical energy into chemical energy, electrocatalytic reactions are vital for closing the anthropogenic carbon cycle. A wide array of electrocatalytic reactions have gained extensive attention, including hydrogen evolution (HER), oxygen evolution (OER), and CO_2_ electroreduction (CO_2_RR). However, progress in enhancing catalyst activity, selectivity, and stability is impeded by the lack of fundamental insight. Under reaction conditions, multi-step electron transfer and elementary catalytic processes convolute, influenced by the applied potential that affects the electrostatic potential of electrons through charging of the electric double layer (EDL) and/or the chemical potential of the electrocatalysts. This complexity presents challenges in interpreting catalytic mechanisms, especially for non-metallic catalysts, where both chemical and electrostatic potentials are altered by the applied voltage. For semiconducting catalysts in electrolytes, it remains a key challenge to resolve how (i) the conduction band and the Fermi level shift, (ii) the potential drops across semiconductor-electrolyte interfaces, and (iii) electron transfer pathways correlate with catalytic rates [1–3]. While conventional electrochemical techniques can only provide ensemble information, thus neglecting the spatial heterogeneity in the electronic structure and catalytic sites of electrode materials, differentiation of potential distributions between non-metallic catalysts and the electric double layer (EDL) relies largely on theoretical calculations.

**Figure 1.**
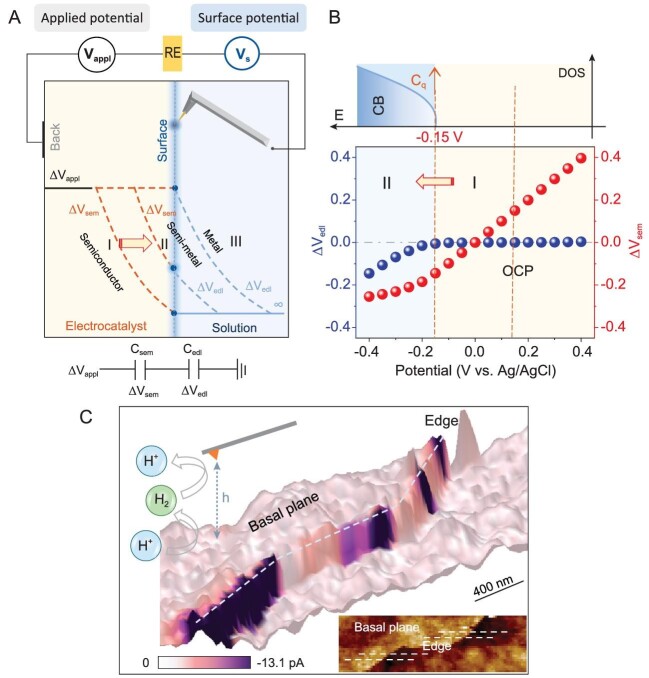
(A) Schematic of the *in situ* surface potential measurement setup and potential drops across the electrode-electrolyte interface for metal, semi-metal, and semiconductor, respectively. (B) Decoupled contribution of potential drops across the monolayer MoS_2_ basal plane–electrolyte interface as a function of the applied potential. (C) HER current imaging on the monolayer MoS_2_ electrocatalyst, superimposed on the corresponding 3D topography map. Adapted and reprinted with permission from Ref. [4].

In an article published in *National Science Review*, Wang *et al.* tackle the intricate task of directly probing the potential distribution across semiconductor catalyst-electrolyte interfaces and disentangling the convoluted processes

of charge transfer and catalytic conversion [4]. They systematically elucidate in liquid phase how the voltage applied to monolayer-thick molybdenum disulfide (MoS_2_) electrocatalysts impacts their electronic structure, the properties of electron transfer across the solid-liquid interface, and the HER activity. To achieve this, the authors employ an innovative method based on *in situ* surface potential microscopy, by integrating a high impedance amplifier with an atomic force microscope (AFM) and atomic force microscopy-based scanning electrochemical microscopy (AFM-SECM), allowing the spatial mapping of both electron transfer and electrocatalytic activity (Fig. 1). Through this approach, the authors were able to decouple the potential drop across the MoS_2_ basal plane and the electrical double layer as a function of the applied voltage. With increasing cathodic potential, they observe the migration of the Fermi level into the conduction band of MoS_2_, which is in contrast to the classical theory saying that the Schottky-analogue junction is broken once the Fermi level is tuned into the conduction band. The authors show that the MoS_2_ surface undergoes a transition from high electron concentration under cathodic potentials to low electron concentration under anodic potentials, leading to the electrolyte gating effect. This is further supported by *in situ* local electric conductivity measurements which reveal that the MoS_2_ surface switches between conductive and insulating state in liquid environments.

Moreover, based on AFM-SECM mapping using outer-sphere redox pairs, the authors find that electron transfer rates at various potentials are similarly high for both the basal plane and edges of MoS_2_. This finding challenges the traditional consensus developed under *ex situ* conditions and is rationalized by the high surface charge concentration occurring *in situ*. By further decoupling the potential-dependent surface charge concentration and electron transfer rate constant for MoS_2_, the authors systematically elucidate three regimes at increasing cathodic potential: (i) transformation from a semiconductor state to a semi-metallic state with increased surface charge density, (ii) gradual potential drop increase within the EDL and creation of a driving force for electrons to transfer from MoS_2_ to solution, with the applied voltage acting on the electric field intensity, and (iii) increasing electron transfer rate constant, with the applied voltage mainly prompting electrons to pass through the solid-liquid interface. Furthermore, the authors analyze *in situ*, at the nanoscale, the activity of MoS_2_ towards inner-sphere electrocatalytic HER. They observe significantly higher HER activity at the edge sites compared to the basal planes. In addition, the HER currents are found to vary at different edge sites, indicating the inner-sphere reaction being more sensitive to the chemical properties of the active sites. By comparing maps of electron transfer and HER activity, the authors deduce that the basal plane's low electrocatalytic activity arises from a lack of active sites rather than its electron transfer capability. While the applied voltage increases the surface electron concentration and electron transfer rate constant of the MoS_2_ basal plane, a large number of electrons reaching the surface cannot participate in the chemical reaction, with the low binding energies of H atoms at basal planes leading to the low HER rates.

The results achieved by combining *in situ* AFM-based characterization techniques mark a significant leap forward in the fundamental understanding of photo-electrocatalytic material systems. The authors unveiled an *in situ* method to spatially discern variations in the chemical potential of electrocatalysts and the electrostatic potential across the EDL, which critically depends on the catalyst's electronic structure. Semiconducting metal oxide catalysts adapt their band structure and surface charge density in response to applied potentials. Altering the redox state of the metal-oxygen bond inherently modifies the chemical potential of the catalyst itself, which was found to dictate the activation energy of the rate-determining step in OER. However, these electronic structure adjustments of heterogeneous solid catalysts must be complemented with highly catalytically active sites to achieve efficient energy conversion. In the case of metal surfaces, the potential predominantly diminishes within the electrode-electrolyte interface, characterized by a strong interfacial electric field. The methods applied in this study present substantial opportunities to analyze inner- and outer-sphere catalytic processes *in situ* at high spatial resolution, facilitating the differentiation between ensemble and local catalyst properties at the nanometer scale [5–7].

In addition, this research opens up avenues for tackling pivotal challenges in complex heterogenous catalytic systems, including the elucidation of corrosion sites and local interfacial chemical properties, to establish reliable structure-property relationships and design next-generation photo-electrocatalysts.
